# Whole-genome sequencing of SARS-CoV-2 from the initial cases of domestic cat infections in Canada

**DOI:** 10.1128/mra.01295-23

**Published:** 2024-02-27

**Authors:** Asma Sultana, Dorothee Bienzle, Scott Weese, Brad Pickering, Peter Kruczkiewicz, Greg Smith, Mathieu Pinette, Oliver Lung

**Affiliations:** 1 Department of Biological Sciences, University of Manitoba, Winnipeg, Manitoba, Canada; 2 National Centre for Foreign Animal Disease, Canadian Food Inspection Agency, Winnipeg, Manitoba, Canada; 3 Ontario Veterinary College, Centre for Public Health and Zoonoses, University of Guelph, Ontario, Guelph, Canada; 4 Department of Medical Microbiology and Infectious Diseases, University of Manitoba, Winnipeg, Manitoba, Canada; DOE Joint Genome Institute, USA

**Keywords:** SARS-CoV2, WGS, cat, pets, coronavirus

## Abstract

Two cat nasal swabs from Canada’s earliest confirmed SARS-CoV-2 positive domestic cats were sequenced to over 99% SARS-CoV-2 genome coverage. One cat had lineage A.23.1 SARS-CoV-2 not reported before in animals. Both sequences have multiple spike gene mutations and clustered closely with human-derived sequences in the global SARS-CoV-2 phylogenetic tree.

## ANNOUNCEMENT

Severe acute respiratory syndrome coronavirus 2 (SARS-CoV-2) (genus *Betacoronavirus*, family *Coronaviridae*), the causative agent of the COVID-19 pandemic, had a profound impact on public health. Natural and experimental infections confirmed the susceptibility of 29 species, including free-living, domestic, captive, and farmed animals (1–6). Natural infections have been reported in nine feline species, including domestic cat (*Felis catus*), tiger (*Panthera tigris*), lynx (*Lynx lynx* and *L. canadensis*), and lion (*P. leo*) (6–8). Furthermore, animal-to-human transmission of SARS-CoV-2 has been reported for hamsters, mink, cats, and white-tailed deer (3, 8–10). Characterization of SARS-CoV-2 in animals improves our understanding of potential intermediate hosts.

From October 2020 to April 2021, the Animal Health Laboratory, University of Guelph, sent 11 samples (oral, nasal, and rectal swabs) from four cats to the National Centre for Foreign Animal Disease for confirmatory testing. RNA was extracted from the swab samples using the MagMax CORE Nucleic Acid Purification Kit (ThermoFisher Scientific), and qRT-PCR targeting the E (3) and RdRp genes [Forward primer-GTGAAATGGTCATGTGTGGCGG, Reverse primer- CAAATGTTAAAAACACTATTAGCATA and probe-FAM/BHQ-1-CAGGTGGAACCTCATCAGGAGATGC) of SARS-CoV-2 was performed (unpublished)].

**Fig 1:**
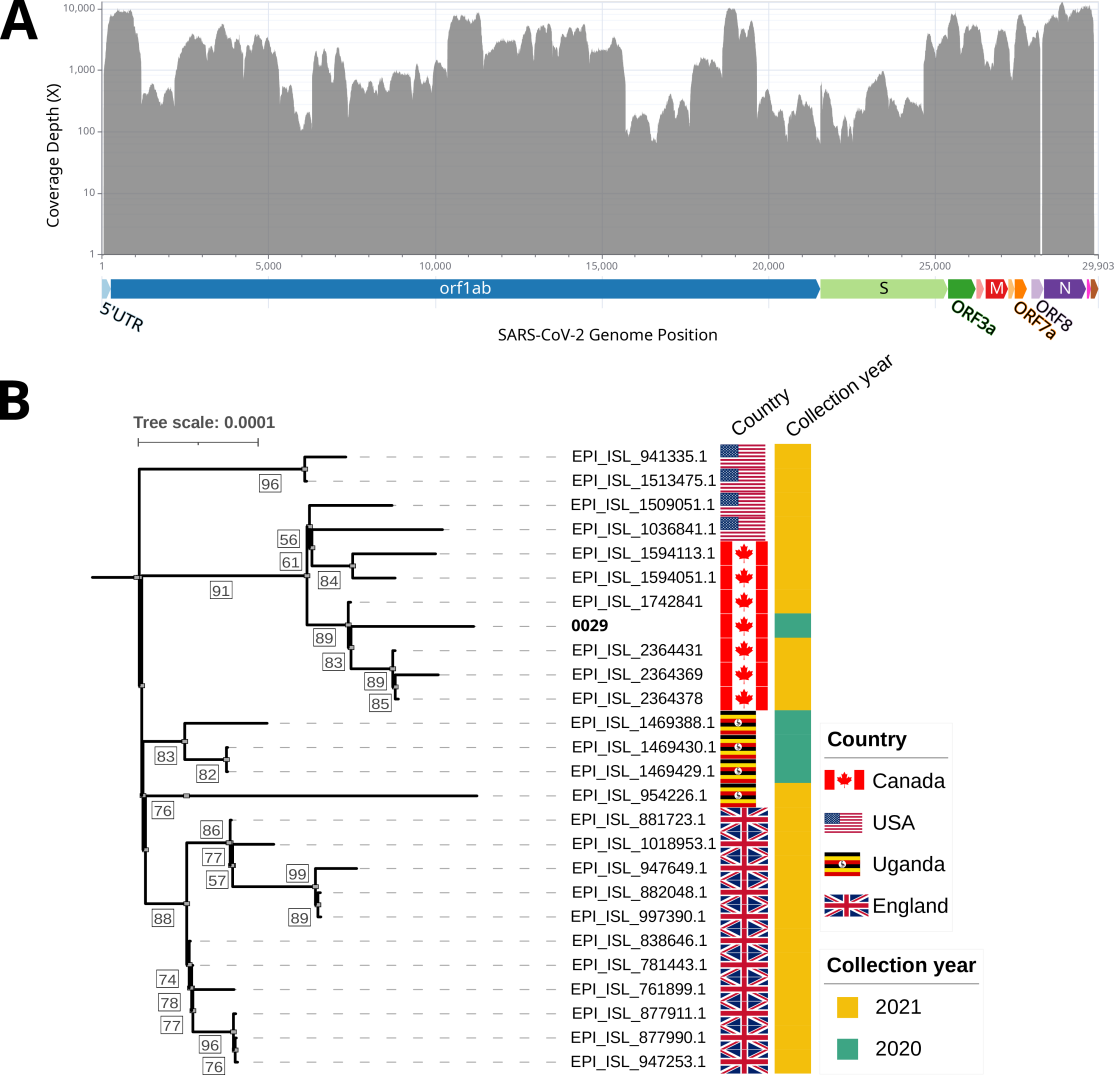
(**A**) A barplot of the sequencing coverage depth across the SARS-CoV-2 genome of the lineage A.23.1 sequence recovered from a Canadian cat (sample WIN-AH-2021-OTH-Kari-0029-OS-1) generated using wgscovplot (https://github.com/nhhaidee/wgscovplot). The x-axis shows the SARS-CoV2 genome position, and the y-axis shows genome coverage depth. At the bottom, the whole genome of the SARS-CoV2 reference strain, including gene features, is attached. (**B**) A maximum-likelihood phylogenetic tree using the whole genome of lineage A.23.1 SARS-CoV-2 sequence from a Canadian cat (sample WIN-AH-2021-OTH-Kari-0029-OS-1; denoted as 0029 in the tree) along with 25 most closely related lineage A.23.1 sequences from GISAID (20) as identified by UShER phylogenetic placement analysis (2023-12-07) which are collected from different geographic regions but at the similar time. Sequence alignment was performed using MAFFT (v7.511) under the default settings (Method- FFT-NS-2) (21), and the maximum-likelihood phylogenetic tree was inferred using IQ-TREE (v1.6.12) with the K3Pu + F model (determined by IQ-TREE’s ModelFinder) and 1,000 ultra-fast bootstraps (22–24). SARS-CoV-2 Wuhan-Hu-1 reference sequence (MN908947.3) has been used as the outgroup.

The qRT-PCR-positive samples (*n* = 9, Ct values 26–36) were amplified using a 1,200 bp tiled PCR amplicon protocol (11). Amplicons for four samples, including sample NCFAD-2020-0085 (0085; sampled in November 2020), were sequenced on a FLO-MIN106 flow cell with a GridION sequencer following library preparation with the Native barcoding (EXP-NBD104) and Ligation sequencing (SQK-LSK109) kits (Oxford Nanopore Technologies) (12). SPRI beads were used for the selection of >1,200 bp fragmented DNA. Basecalling, barcode demultiplexing, adapter trimming, and read quality control were performed with Guppy (v4.0.11) using the high-accuracy model. 1.6M reads were generated and the estimated N50 was 1.24 kb. Error correction was not performed since a high allele fraction threshold of 75% was selected for calling high-confidence variants from read alignments. Amplicons for the other five samples, including sample WIN-AH-2021-OTH-Kari-0029-OS-1 (0029) from Ontario, were sequenced on an Illumina MiSeq after processing with the Nextera XT DNA kit, producing 150 bp paired-end reads. Nanopore and Illumina sequencing reads were analyzed with the Nextflow (v23.10.0) (13) pipelines, CFIA-NCFAD/nf-virontus (v2.0.0dev1) (14), and nf-core/viralrecon (v2.6.0) (15, 16), respectively, using SARS-CoV-2 Wuhan-Hu-1 reference sequence (MN908947.3). Nextclade was used to find mutations from the consensus sequence. Over 99% of the genomes were recovered from 0029 and 0085, with 2543.7X and 14,164X depth of coverage, respectively (Fig 1).

Pangolin (v4.2) (17) classified sample 0085 as lineage B.1.2 and 0029as lineage A.23.1. The A.23.1 lineage was first reported in Uganda in late 2020 (18) but has never been reported in animals (GISAID and SARS-ANI VIS database search on 2024-01-24) 26 synonymous and non-synonymous mutations were present in 0029, whereas 22 mutations were identified in 0085 (Table 1). Phylogenetic placement analysis with UShER (19) using 16,490,767 SARS-CoV-2 sequences from GISAID, GenBank, COG-UK, and CNCB (2023-12-05) revealed that the human-derived SARS-CoV-2 sequence Canada/2021/EPI_ISL_1742841 (lineage A.23.1; Fig. 1) was the most closely related to 0029 while USA/2020/MZ908099.1 (lineage B.1.2) was the most closely related human derived sequence to 0085. Default parameters were used for all data analysis software.

**TABLE 1:** The non-synonymous mutations observed in cat-derived SARS-CoV-2 sequences WIN-AH-2021-OTH-Kari-0029-OS-1 and NCFAD-2020-0085 relative to the Wuhan-Hu-1 reference sequence (MN908947.3)

Sample	Gene	Nucleotide mutation	Amino acid mutation
WIN-AH-2021-OTH-Kari-0029-OS-1	ORF1ab	G1820A	G519S
(29,655 nucleotides) (38% GC content)	ORF1ab	C10038T	T3258I
	ORF1ab	G10540A	M3425I
	ORF1ab	G11230T	M3655I
	ORF1ab	G11266T	L3667F
	ORF1ab	G11521T	M3752I
	ORF1ab	C16575T	T5437I
	ORF1ab	C17745T	T5827I
	ORF1ab	A18102G	H5946R
	S	G21777A	G72E
	S	G21867T	R102I
	S	C22033A	F157L
	S	G22661T	V367F
	S	G23401T	Q613H
	S	C23604G	P681R
	ORF8	T28144C	L84S
	N	G28307A	A12T
	N	G28878A	S202N
NCFAD-2020-0085	ORF1a	C1059T	T265I
(29,786 nucleotides) (38% GC content)	ORF1a	G8083A	M2606I
	ORF1a	C10319T	L3352F
	ORF1b	C14407T	P314S
	ORF1b	C14408T	P314L
	ORF1b	A18424G	N1653D
	ORF1b	C21304T	R2613C
	S	A23403G	D614G
	S	G23593T	Q677H
	ORF3a	G25563T	Q57H
	ORF3a	G25907T	G172V
	M	G26775T	A85S
	ORF8	C27964T	S24L
	N	C28472T	P67S
	N	C28869T	P199L
	N	G29402T	D377Y

## Data Availability

These two cat SARS-CoV-2 genomes (WIN-AH-2021-OTH-Kari-0029-OS-1 and NCFAD-2020-0085) were deposited in the GenBank (accession numbers: OR999078.1 and OR999071.1). The accession numbers for the Illumina MiSeq and Oxford Nanopore GridION sequencing raw reads in the NCBI Sequence Read Archive (SRA) are PRJNA1055551 (BioProject), SRR27318679 (SRA), and SAMN39052760 (BioSample) for WIN-AH-2021-OTH-Kari-0029-OS-1 sample and PRJNA1055563 (BioProject), SRR27319240 (SRA), and SAMN39053918 (BioSample) for NCFAD-2020-0085 sample.

## References

[1] Segalés J, Puig M, Rodon J, Avila-Nieto C, Carrillo J, Cantero G, Terrón MT, Cruz S, Parera M, Noguera-Julián M, Izquierdo-Useros N, Guallar V, Vidal E, Valencia A, Blanco I, Blanco J, Clotet B, Vergara-Alert J. 2020. Detection of SARS-CoV-2 in a cat owned by a COVID-19−affected patient in Spain. Proc Natl Acad Sci USA 117:24790–24793. doi:10.1073/pnas.2010817117 32948692 PMC7547282

[2] Sit THC, Brackman CJ, Ip SM, Tam KWS, Law PYT, To EMW, Yu VYT, Sims LD, Tsang DNC, Chu DKW, Perera RAPM, Poon LLM, Peiris M. 2020. Infection of dogs with SARS-CoV-2. Nature 586:776–778. doi:10.1038/s41586-020-2334-5 32408337 PMC7606701

[3] Pickering B, Lung O, Maguire F, Kruczkiewicz P, Kotwa JD, Buchanan T, Gagnier M, Guthrie JL, Jardine CM, Marchand-Austin A, et al.. 2022. Divergent SARS-CoV-2 variant emerges in white-tailed deer with deer-to-human transmission. Nat Microbiol 7:2011–2024. doi:10.1038/s41564-022-01268-9 36357713 PMC9712111

[4] Sia SF, Yan L-M, Chin AWH, Fung K, Choy K-T, Wong AYL, Kaewpreedee P, Perera RAPM, Poon LLM, Nicholls JM, Peiris M, Yen H-L. 2020. Pathogenesis and transmission of SARS-CoV-2 in golden hamsters. Nature 583:834–838. doi:10.1038/s41586-020-2342-5 32408338 PMC7394720

[5] Shi J, Wen Z, Zhong G, Yang H, Wang C, Huang B, Liu R, He X, Shuai L, Sun Z, Zhao Y, Liu P, Liang L, Cui P, Wang J, Zhang X, Guan Y, Tan W, Wu G, Chen H, Bu Z. 2020. Susceptibility of ferrets, cats, dogs, and other domesticated animals to SARS–coronavirus 2. Science 368:1016–1020. doi:10.1126/science.abb7015 32269068 PMC7164390

[6] Nerpel A, Käsbohrer A, Walzer C, Desvars-Larrive A. 2023. Data on SARS-CoV-2 events in animals: mind the gap!. One Health 17:100653. doi:10.1016/j.onehlt.2023.100653 38024278 PMC10665207

[7] Qiu X, Liu Y, Sha A. 2023. SARS‐CoV‐2 and natural infection in animals. J Med Virol 95:e28147. doi:10.1002/jmv.28147 36121159 PMC9538246

[8] Yen H-L, Sit THC, Brackman CJ, Chuk SSY, Gu H, Tam KWS, Law PYT, Leung GM, Peiris M, Poon LLM, Cheng SMS, Chang LDJ, Krishnan P, Ng DYM, Liu GYZ, Hui MMY, Ho SY, Su W, Sia SF, Choy K-T, Cheuk SSY, Lau SPN, Tang AWY, Koo JCT, Yung L. 2022. Transmission of SARS-CoV-2 delta variant (AY.127) from pet hamsters to humans, leading to onward human-to-human transmission: a case study. Lancet 399:1070–1078. doi:10.1016/S0140-6736(22)00326-9 35279259 PMC8912929

[9] Ghai RR, Straily A, Wineland N, Calogero J, Stobierski MG, Signs K, Blievernicht M, Torres-Mendoza Y, Waltenburg MA, Condrey JA, et al.. 2023. Epidemiologic and genomic evidence for zoonotic transmission of SARS-CoV-2 among people and animals on a Michigan mink farm, United States, 2020. Viruses 15:2436. doi:10.3390/v15122436 38140677 PMC10747742

[10] Sila T, Sunghan J, Laochareonsuk W, Surasombatpattana S, Kongkamol C, Ingviya T, Siripaitoon P, Kositpantawong N, Kanchanasuwan S, Hortiwakul T, Charernmak B, Nwabor OF, Silpapojakul K, Chusri S. 2022. Suspected cat-to-human transmission of SARS-CoV-2, Thailand, July–September 2021. Emerg Infect Dis 28:1485–1488. doi:10.3201/eid2807.212605 35666777 PMC9239874

[11] Freed NE, Vlková M, Faisal MB, Silander OK. 2020. Rapid and inexpensive whole-genome sequencing of SARS-CoV-2 using 1200 bp tiled amplicons and Oxford nanopore rapid barcoding. Biol Methods Protoc 5:bpaa014. doi:10.1093/biomethods/bpaa014 33029559 PMC7454405

[12] Fisher M, Nebroski M, Davies J, Janzen E, Sullivan D, Lung O. 2023. Discovery and comparative genomic analysis of a novel equine anellovirus, representing the first complete mutorquevirus genome. Sci Rep 13:3703. doi:10.1038/s41598-023-30875-7 36878942 PMC9988894

[13] Di Tommaso P, Chatzou M, Floden EW, Barja PP, Palumbo E, Notredame C. 2017. Nextflow enables reproducible computational workflows. Nat Biotechnol 35:316–319. doi:10.1038/nbt.3820 28398311

[14] Kruczkiewicz P, Lung O. 2023. CFIA-NCFAD/Nf-Virontus V2.0.0Dev1 (2.0.0Dev1). Zenodo. doi:10.5281/zenodo.10626229

[15] Patel H, Monzón S, Varona S, Espinosa-Carrasco J, Garcia MU, Heuer ML, Underwood A, Gabernet G, Ewels P, MiguelJulia K, S T, H F, Wilson S, Erika S, K W, M jcurado, Menden K. 2023. Nf-core/Viralrecon: Nf-core/Viralrecon V2.6.0 - Rhodium Raccoon (2.6.0). Zenodo. doi:10.5281/zenodo.7764938

[16] Ewels PA, Peltzer A, Fillinger S, Patel H, Alneberg J, Wilm A, Garcia MU, Di Tommaso P, Nahnsen S. 2020. The nf-core framework for community-curated bioinformatics pipelines. Nat Biotechnol 38:276–278. doi:10.1038/s41587-020-0439-x 32055031

[17] O’Toole Á, Scher E, Underwood A, Jackson B, Hill V, McCrone JT, Colquhoun R, Ruis C, Abu-Dahab K, Taylor B, Yeats C, du Plessis L, Maloney D, Medd N, Attwood SW, Aanensen DM, Holmes EC, Pybus OG, Rambaut A. 2021. Assignment of epidemiological lineages in an emerging pandemic using the pangolin tool. Virus Evol 7:veab064. doi:10.1093/ve/veab064 34527285 PMC8344591

[18] Bugembe DL, Phan MVT, Ssewanyana I, Semanda P, Nansumba H, Dhaala B, Nabadda S, O’Toole ÁN, Rambaut A, Kaleebu P, Cotten M. 2021. Emergence and spread of a SARS-CoV-2 lineage a variant (A.23.1) with altered spike protein in Uganda. Nat Microbiol 6:1094. doi:10.1038/s41564-021-00933-9 34163035 PMC8318884

[19] Turakhia Y, Thornlow B, Hinrichs AS, De Maio N, Gozashti L, Lanfear R, Haussler D, Corbett-Detig R. 2021. Ultrafast sample placement on existing tRees (UShER) enables real-time phylogenetics for the SARS-CoV-2 pandemic. Nat Genet 53:809–816. doi:10.1038/s41588-021-00862-7 33972780 PMC9248294

[20] Shu Y, McCauley J. 2017. GISAID: global initiative on sharing all influenza data – from vision to reality. Euro Surveill 22:30494. doi:10.2807/1560-7917.ES.2017.22.13.30494 28382917 PMC5388101

[21] Katoh K, Standley DM. 2013. MAFFT multiple sequence alignment software version 7: improvements in performance and usability. Mol Biol Evol 30:772–780. doi:10.1093/molbev/mst010 23329690 PMC3603318

[22] Nguyen L-T, Schmidt HA, von Haeseler A, Minh BQ. 2015. IQ-TREE: a fast and effective stochastic algorithm for estimating maximum-likelihood phylogenies. Mol Biol Evol 32:268–274. doi:10.1093/molbev/msu300 25371430 PMC4271533

[23] Kalyaanamoorthy S, Minh BQ, Wong TKF, von Haeseler A, Jermiin LS. 2017. ModelFinder: fast model selection for accurate phylogenetic estimates. Nat Methods 14:587–589. doi:10.1038/nmeth.4285 28481363 PMC5453245

[24] Minh BQ, Nguyen MAT, von Haeseler A. 2013. Ultrafast approximation for phylogenetic bootstrap. Mol Biol Evol 30:1188–1195. doi:10.1093/molbev/mst024 23418397 PMC3670741

